# The Relationship between Routine Blood Parameters and the Prognosis of COVID-19 Patients in the Emergency Department

**DOI:** 10.1155/2021/7489675

**Published:** 2021-12-01

**Authors:** Birsen Ertekin, Mehmet Yortanlı, Ozan Özelbaykal, Ali Doğru, A. Sadık Girişgin, Tarık Acar

**Affiliations:** ^1^Department of Emergency, Beyhekim Training and Research Hospital, Konya, Turkey; ^2^Department of Emergency, Numune State Hospital, Konya, Turkey; ^3^Department of Emergency, Necmettin Erbakan University Meram Faculty of Medicine, Konya, Turkey

## Abstract

The aim of this study is to investigate the routine blood parameters of COVID-19 patients at the time of admission to the emergency department and their relationship with the severity of the disease and prognosis. A total of 500 patients, who were diagnosed with severe COVID-19 and hospitalized in the intensive care unit between 01.04.2020 and 01.02.2021 in the emergency department of a pandemic hospital, were retrospectively analyzed. Demographic, clinical, and laboratory data of the patients were obtained from the hospital registry system. Neutrophil-to-lymphocyte ratio (NLR), monocyte-to-lymphocyte ratio (MLR), and platelet-to-lymphocyte ratio (PLR) were calculated using neutrophil, lymphocyte, monocyte, and platelet counts. These patients were divided into two groups: survivors and deceased. All parameters obtained from routine blood analysis were statistically compared between these two groups. While 280 out of 500 patients survived, 220 died. Of all patients, the mean age was 67 years and 51.8% were males. There was a significant difference between the two groups in terms of age, gender, length of hospital stay, need for mechanical ventilation, white blood cell, neutrophil, lymphocyte, monocyte, eosinophil, platelet counts, CRP, ferritin, procalcitonin values, NLR, MLR, and PLR (*p* < 0.001 for all). While NLR alone and MLR + NEU and NLR + PLR + MLR combinations had the highest AUC values (0.930, 0.947, and 0.939, respectively), MLR and PLR alone showed the lowest AUC values (0.875 and 0.797, respectively). The sensitivity, specificity, positive predictive values (PPVs), and negative predictive values (NPVs) in the prediction of death according to the cutoff values of the parameters have been determined. A significant correlation was determined between age, NLR, MLR, and PLR and duration of hospital stay (*p* < 0.001 for all). Routine blood parameters and NLR, MLR, and PLR can assist emergency physicians to identify the severity and early prognosis of COVID-19 patients.

## 1. Introduction

Coronavirus disease 2019 (COVID-19) caused by severe acute respiratory syndrome 2 (SARS-CoV-2) has become an important health problem worldwide [1]. COVID-19 usually begins with flu-like symptoms [2]. However, a certain percent of patients may suffer from severe course of the infection [3]. COVID-19, which has rapidly spread worldwide, may lead to asymptomatic infection, viral pneumonia, acute respiratory distress syndrome (ARDS), multiple organ dysfunction syndrome (MODS), shock, and even death [4]. Early diagnosis and treatment of COVID-19 are of vital importance given its rapid spread and severe complications [5]. Therefore, early detection of the factors that may lead to death would improve the prognosis through enabling early intervention [6]. Recent studies indicate that severe COVID-19 patients may have immune dysregulation that leads to the development of viral hyperinflammation. This hyperinflammatory response may result in MODS and death by causing cytokine storm [7, 8]. All COVID-19 patients should be screened for hyperinflammation by using laboratory parameters in order to decrease mortality [9]. Various abnormal hematological parameters including leukocytosis, neutrophilia, thrombocytopenia, lymphopenia, elevated CRP, procalcitonin, D-dimer, and fibrinogen levels have been shown in many studies conducted with COVID-19 patients [10, 11]. Neutrophil-to-lymphocyte ratio (NLR) is considered as an inflammatory marker and has been found to be increased in various conditions such as sepsis [12], metabolic syndrome [13], pulmonary embolism [14], and malignancy [15]. It is also associated with COVID-19 infection [5]. Similarly, platelet-to-lymphocyte ratio (PLR) has been introduced as a marker of inflammatory diseases including pulmonary embolism [14], SARS-CoV-2 infection [16], and cancer [17]. Increased monocyte-to-lymphocyte ratio (MLR) values were reported in subjects with COVID-19 infection [18], liver inflammation [19], and rheumatoid arthritis [20]. Since COVID-19 infection is associated with increased inflammatory burden, these parameters might also be related to severe COVID-19 infection. In the present study, it was investigated whether or not routine peripheral blood parameters and NLR, MLR, and PLR, which are obtained from those parameters, have a relationship with the prognosis of COVID-19 patients in the emergency department.

## 2. Materials and Methods

### 2.1. Patients and Process

A total of 500 patients, who had been diagnosed with severe COVID-19 and hospitalized in the intensive care unit between 01.04.2020 and 01.02.2021 in the emergency department of a pandemic hospital and who fulfilled the inclusion criteria, were retrospectively analyzed. The following criteria were considered for the diagnosis of severe COVID-19 pneumonia: (1) fever and respiratory tract infection findings and/or (2) respiratory rate > 30/min and/or (3) severe respiratory distress (dyspnea, tachypnea, and use of extra respiratory muscles) and/or (4) oxygen saturation in room air of <90% (PaO_2_/FiO_2_ ≤ 300 in the patient receiving oxygen) and/or (5) the presence of bilateral lobular, peripherally located, diffusely patched ground-glass opacities that are the characteristic findings of COVID-19 pneumonia on the computed tomography of the thorax [21]. Patients whose spiral computed tomography (CT) of the thorax report was approved by a radiology and chest diseases specialist and who had undergone real-time reverse transcriptase-polymerase chain reaction (RT-PCR) at least twice at 24-hour intervals, at least one of which was positive, were included in the study. In addition, the diagnosis of COVID-19 in those without typical radiological findings was made by an infectious diseases specialist based on clinical features, laboratory results, and radiological appearances, without an alternative diagnosis [22]. Patients under the age of 18, pregnant women, those with chronic obstructive pulmonary disease and hematological disease and cancers, immunosuppressive patients, those who had been exposed to trauma, and those whose information could not be accessed from the electronic registry system were excluded from the study. Age, gender, medical history, clinical and physical examination findings of these patients, peripheral routine blood analysis (white blood cell (WBC) count, neutrophil, lymphocyte, monocyte, eosinophil, platelet counts, CRP, D-dimer, ferritin, and procalcitonin (PRC) values) at the time of admission to the emergency department, the PCR result, report of CT of the thorax, need for mechanical ventilation (noninvasive/invasive/high-flow nasal cannula oxygen), total duration of hospital stay, and clinical outcomes (discharge/death) were obtained retrospectively from the hospital's registry system. The neutrophil-to-lymphocyte ratio (NLR), the monocyte-to-lymphocyte ratio (MLR), and the platelet-to-lymphocyte ratio (PLR) were calculated using neutrophil, lymphocyte, monocyte, and platelet counts obtained from the blood analysis. These patients were divided into two groups: survivors and deceased. All parameters obtained from the routine blood analysis were statistically compared between these two groups. In addition, the correlation between age, NLR, MLR, and PLR and the duration of hospital stay was evaluated. The study was approved by the Local Ethics Committee of Necmettin Erbakan University Faculty of Medicine (date: 19/03/2021 and number: 2021/3167) and was conducted in accordance with the ethical principles of the Declaration of Helsinki.

### 2.2. Statistical Analysis

A descriptive analysis was performed. Categorical data were given as ratios and numbers. They were compared using the chi-square test. The distribution of the numerical data was examined by visual and analytical methods. There were no normally distributed variables, and nonnormally distributed variables were given as median and interquartile range (IQR). The differences between survivors and nonsurvivors were compared using the Mann–Whitney *U* test for nonnormally distributed variables. Correlation coefficients and statistical significance were calculated with Spearman's test for the relationships between the duration of hospital stay, NLR, MLR, PLR, and age. Possible factors for predicting mortality were analyzed using a multiple logistic regression model. Multiple regression modeling was applied by applying backward variable selection to evaluate the relationship with each of the results. The diagnostic decision-making properties of NLR, MLR, PLR, and new models in predicting mortality were analyzed using the receiver operating characteristic (ROC) curve analysis. In the presence of significant breakpoints, the sensitivity, specificity, PPVs, and NPVs of these limits were calculated. In the evaluation of the area under the curve, the cases where the type 1 error level was below 5% were interpreted as the diagnostic value of the test, which was statistically significant. Cases with a *p* value below 0.05 were considered statistically significant. Statistical analysis was calculated using the IBM SPSS 26 program.

## 3. Results

The comparison of two groups (survivors and deceased) with regard to demographic, clinical, and laboratory data is shown in Table 1. While 280 out of 500 patients survived, 220 died. Of all patients, the mean age was 67 years (IQR 25) and 259 (51.8%) were males. A significant difference was determined between the groups with regard to age, gender, duration of hospital stay, need for mechanical ventilation, computed tomography of the thorax findings, routine blood parameters (WBC, neutrophil, lymphocyte, monocyte, eosinophil, platelet counts, CRP, ferritin, and PRC values), NLR, MLR, and PLR (*p* < 0.001 for all).

**Figure 1:**
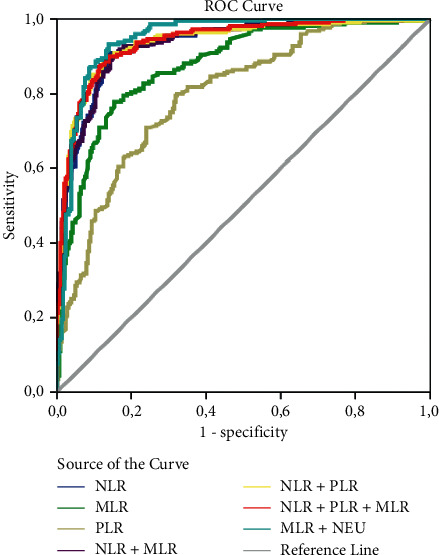
The ROC curves of parameters NLR, MLR, and PLR in the diagnosis of severe COVID-19 on admission. NLR + MLR: the integration parameters of MLR and NLR; NLR + PLR: the integration parameters of PLR and NLR; NLR + MLR + PLR: the integration parameters of PLR, MLR, and NLR; MLR + NEU: the integration parameters of MLR and neutrophils.

The ROC analyses of NLR, MLR, and PLR, which were obtained from routine blood parameters obtained at the time of admission, are presented in Figure 1. The AUC values of these parameters are displayed in Table 2. According to Table 2, while NLR alone and MLR + NEU and NLR + PLR + MLR combinations had the highest AUC values (0.930, 0.947, and 0.939, respectively), MLR and PLR values alone showed the minimum AUC values (0.875 and 0.797, respectively).

The sensitivity, specificity, positive predictive values (PPVs), and negative predictive values (NPVs) in the prediction of death according to the cutoff values of the parameters have been demonstrated in Table 3. While NLR alone and NLR + MLR and MLR + NEU combinations had the highest sensitivity and NPVs (90.9, 90.9, 93.2–92.3, 92.2, and 94.1, respectively), MLR and PLR alone showed the lowest sensitivity and NPVs (77.7, 79.5–82.6, and 80.9, respectively). A significant correlation was determined between age, NLR, MLR, and PLR and duration of hospital stay (*p* < 0.001 for all) (Table 4).

## 4. Discussion

Severe COVID-19 infection is characterized by an intense proinflammatory response, cytokine storm, and activation of the coagulation cascade that causes ARDS, MODS, and even death [23]. Many infectious diseases cause inflammation, including COVID-19 pneumonia [24]. Severe inflammatory responses contribute to the weakening of the adaptive immune response, and an immune response imbalance occurs. Therefore, laboratory findings that may indicate an inflammatory state are potential predictors of the prognosis of COVID-19 patients [25]. Hematological markers used to classify COVID-19 patients include WBC, lymphocyte, neutrophil, platelet, eosinophil, monocyte count, NLR, MLR, PLR, and hemoglobin. While CRP, ferritin, and PRC are inflammatory markers, D-dimer is among the coagulation markers. These markers not only predict the prognosis but also classify COVID-19 patients into risk categories [26].

There is growing evidence to support that inflammation caused by infectious diseases plays an important role in the progression of various viral cases of pneumonia, including COVID-19 [27]. Increases in many cytokines, especially CRP, sedimentation, ferritin, PRC, and IL-6, have been reported in COVID-19 patients. While the increase in PRC mostly indicates an accompanying secondary bacterial infection, it was found to be associated with the severity of the disease and mortality [28]. Elevated D-dimer has been reported as a poor prognostic factor in COVID-19 patients who have frequent coagulation disorders [29]. Elevated WBC is common in critically ill patients because damaged cells lead to inflammation in the lungs mediated largely by proinflammatory macrophages and granulocytes [30]. Henry et al. reported that elevated WBC levels resulted from elevated neutrophils and decreased lymphocyte, monocyte, and eosinophil counts and increased the risk of mortality [31]. Consistent with the literature, the significantly higher CRP, ferritin, PRC, D-dimer, and WBC values of the deceased patient group in our study indicate that inflammation and coagulation parameters are important prognostic parameters in severe COVID-19 patients. It should also be taken into account that lymphopenia and impaired immune response may make these patients more susceptible to secondary bacterial infections.

Dysregulated immune cell responses are believed to play an important role in the severity of virus-induced diseases. Many studies have reported that patients with severe COVID-19 have higher neutrophil levels compared to other patients [7, 32]. Lymphopenia and overactivation of the inflammatory cascade are important features of COVID-19 and have high prognostic value [33]. While lymphopenia is a risk factor for serious illness and death in COVID-19 patients, lymphocytosis is indicative of better outcomes [34, 35]. During the acute phase of virus-induced infection, eosinophils accumulate in infected tissues to resist infection, resulting in a reduction in eosinophils in peripheral blood. Sun et al. stated that eosinophils were significantly decreased in COVID-19 patients at the time of admission and increased gradually in patients in the intensive care unit (ICU) only after the seventh day of hospitalization [18]. Du et al. also stated that 81.2% of the patients had very low eosinophil counts at the time of admission, which may indicate a poor prognosis [36]. In our study, while neutrophil levels were higher in the deceased patient group than in the survivors, lymphocyte and eosinophil levels were found to be lower (*p* < 0.05). Therefore, these changes in neutrophil, lymphocyte, and eosinophil levels can be used as early warning indicators for triage and follow-up of critically ill patients.

NLR, which is easily calculated by dividing the absolute neutrophil count by the absolute lymphocyte count, has been shown as an inflammatory marker that can predict the probability of death in various studies [12, 37]. The inflammatory response can stimulate the production of neutrophils and accelerate the apoptosis of lymphocytes. Thus, an increase in neutrophil count and a decrease in lymphocyte count cause an increase in NLR [38]. Liu et al. emphasized that NLR levels were an independent risk factor for mortality, especially in male patients with COVID-19, and may help distinguish high-risk individuals [39]. Yang et al. stated that while the cutoff value of NLR was 3.3, the specificity, sensitivity, and AUC values were 0.636, 0.88, and 0.841, respectively and showed a superior prognostic probability in determining the severity of the disease [5]. Sun et al. also stated that NLR was more significant than the other two combination parameters, MLR and PLR [18]. In our study, the NLR was found to be higher in patients who died compared to surviving patients (*p* < 0.001). In addition, NLR alone was found to have higher AUC, sensitivity, and NPVs compared to MLR and PLR. According to these data, we think that NLR levels alone have a superior predictive ability in assessing the prognosis and severity of COVID-19 patients.

According to the current view, the monocyte/macrophage population plays a profound role in the immunopathogenesis of both systemic and visceral hyperinflammatory manifestations of severe COVID-19 [40]. Mehta et al. reported that lymphocytes and monocytes were low in severe COVID-19 patients, and this may be due to the low immune response in patients hospitalized in the ICU because these parameters gradually increase in the following days in patients who are not hospitalized in the ICU [8]. Yang et al. reported that the MLR values of severe COVID-19 patients were significantly lower than those of nonsevere patients, but MLR cannot be used as a potential diagnostic marker due to its AUC value of less than 0.50 [5]. Sun et al. also stated in their study that NLR, MLR, and PLR parameters in the patient group were significantly higher than those in the control group. In addition, they stated that when the NLR and MLR values were combined for the diagnostic efficacy analysis of severe COVID-19, the AUC reached 0.925 with higher sensitivity and specificity [18]. Consistent with these two studies, the MLR level in our study had lower AUC, sensitivity, and NPV levels when evaluated alone, while NLR reached higher prognostic values when combined with PLR, especially the neutrophil levels. For this reason, we believe that it would be more appropriate to use MLR together with other blood parameters in risk classification and predict the prognosis of severe COVID-19 patients.

The changes in the number and activity of platelets can be used as sensitive indicators to reflect the immune response of the body [41]. In a study, it was shown that direct invasion of the coronavirus into the bone marrow causes hematopoietic inhibition, while thrombocytopenia may be associated with lung damage [42]. The thrombocytosis observed in COVID-19 patients and the length of the average hospital stay may be related to the cytokine storm [16]. PLR, an indicator of inflammation, originates mainly from megakaryocytes and plays an important role in thrombosis. It plays a crucial role in the inflammatory response to recruit neutrophils and other inflammatory cells to the injury site [43]. PLR, which is calculated by the ratio of the absolute platelet count to the lymphocyte count, is reported to be a reliable marker in the diagnosis of immune-mediated, metabolic, prothrombotic, and neoplastic diseases [16]. PLR fluctuations are related to immune-inflammatory reactions and are positively associated with another systemic inflammation marker, the NLR [44]. Qu et al. stated that while the cutoff value of PLR was 126.7 in COVID-19 patients, the sensitivity was 100%, the specificity was 81.5%, and when the cutoff value was greater than 126.7, the duration of hospital stay and mortality increased [16]. On the contrary, Jimeno et al. also argued that PLR was not associated with mortality or severe clinical course in COVID-19 patients [45]. As in the study of Yang et al. [5], although the PLR level of our deceased patient group was significantly higher than that of the survivors, PLR levels alone had lower AUC levels compared to NLR and MLR. In our study, the PLR level reached the desired high AUC values only when combined with other parameters such as NLR and MLR. Therefore, PLR can be used alone in severe COVID-19 patients and does not seem to be a strong prognostic marker.

## 5. Limitations

Our study had some limitations. First, our study was single-centered, retrospective, and observational, and the validity of the data recorded from the hospital electronic registry system had not been externally verified. Therefore, it should be confirmed by larger and multicenter studies. Second, dynamic monitoring of blood parameters may have important clinical value for assessing disease progression and treatment efficacy, but we did not follow the change of blood parameters after admission because the aim of our study was to determine the risk classification of COVID-19 patients and to predict the prognosis of the disease, particularly by using routine blood parameters obtained at the time of admission in emergency services. Third, many cytokines including IL-1, IL-6, IL-12, and TNF-alpha increase in COVID-19 infection and may lead to poor outcomes. However, cytokines could not be tested as they are not studied as routine blood parameters in emergency service laboratories in our country.

## 6. Conclusion

Routine blood parameters examined at the time of admission to the emergency department are extremely important in predicting the prognosis of severe COVID-19 patients. The results of this study support that NLR could be a useful predictive parameter for COVID-19 patients. However, MLR and PLR have low prognostic values when used alone; thus, they can only be used in combination with other markers.

## Figures and Tables

**Table 1:** Comparison of demographic, clinical, and laboratory findings of survived and died patients.

Variables	All patients (*n* = 500)	Survived patients (*n* = 280)	Died patients (*n* = 220)	*p* value

Age, median (IQR) (years)	67 (25)	56.5 (22)	80 (18)	<0.001 ^*∗*^
Male, *n* (%)	259 (51.8)	128 (49.4)	131 (50.6)	0.002 ^*∗*^
Female, *n* (%)	241 (48.2)	152 (63.1)	89 (36.9)	
WBC, median (IQR) (10^3^/mL)	9.35 (6.38)	7.95 (5.39)	10.9 (7.6)	<0.001 ^*∗*^
Neutrophil, median (IQR) (10^3^/mL)	7.7 (9.5)	5.5 (2.9)	14.9 (7.85)	<0.001 ^*∗*^
Lymphocyte, median (IQR) (10^3^/mL)	0.68 (0.6)	0.83 (0.54)	0.4 (0.4)	<0.001 ^*∗*^
Monocyte, median (IQR) (10^3^/mL)	0.6 (0.61)	0.4 (0.44)	0.9 (0.56)	<0.001 ^*∗*^
Eosinophil, median (IQR) (10^3^/mL)	0.01 (0.04)	0 (0.02)	0.03 (0.03)	<0.001 ^*∗*^
Platelet, median (IQR) (10^3^/mL)	220 (109)	212 (115)	236.5 (113)	<0.001 ^*∗*^
CRP, median (IQR) (mg/L)	100 (120)	77 (108)	129 (139)	<0001 ^*∗*^
PRC, median (IQR) ( *μ*g/L)	0.2 (0.7)	0.2 (0.4)	0.4 (1.4)	<0.001 ^*∗*^
Ferritin, median (IQR) ( *μ*g/L)	320 (573)	247 (529)	398.5 (615)	<0.001 ^*∗*^
D-dimer, median (IQR) (*μ*g/mL)	1.2 (6.8)	0.9 (3.6)	2 (8.6)	0.003 ^*∗*^
MV support, *n* (%)	250 (50)	57 (22.8)	193 (77.2)	<0.001 ^*∗*^
Consolidation in CT, *n* (%)	439 (87.8)	232 (52.8)	207 (47.2)	<0.001 ^*∗*^
Length of stay in hospital, median (IQR) (days)	12 (14)	6 (5)	22 (12)	<0.001 ^*∗*^
NLR, median (IQR)	11.17 (27.77)	6.75 (5.81)	35.53 (34.42)	<0.001 ^*∗*^
MLR, median (IQR)	0.82 (1.53)	0.5 (0.54)	1.86 (2.08)	<0.001 ^*∗*^
PLR, median (IQR)	366.9 (367.8)	264.4 (233.3)	548.3 (469.5)	<0.001 ^*∗*^

WBC: leucocyte; PRC: procalcitonin; MV: mechanical ventilation; CT: spiral computed tomography; NLR: neutrophil-to-lymphocyte ratio; MLR: monocyte-to-lymphocyte ratio; PLR: platelet-to-lymphocyte ratio.

**Table 2:** Area under the receiver operating characteristics curve (AUROC) for the value of NLR, MLR, and PLR in predicting severe COVID-19 mortality.

Parameters	AUC	Mortality 95% confidence interval	*p* value

NLR	0.930	0.908–0.952	<0.001^*∗*^
MLR	0.875	0.844–0.905	<0.001^*∗*^
PLR	0.797	0.758–0.835	<0.001^*∗*^
NLR + MLR	0.931	0.910–0.953	<0.001^*∗*^
NLR + PLR	0.936	0.914–0.958	<0.001^*∗*^
NLR + PLR + MLR	0.939	0.918–0.959	<0.001^*∗*^
MLR + NEU	0.947	0.928–0.967	<0.001^*∗*^

**Table 3:** The value of NLR, MLR, and PLR in diagnosis of severe COVID-19 on admission.

	Cutoff	Sensitivity (%)	Specificity (%)	PPV (%)	NPV (%)

NLR	≥12.1	90.9	85.4	83	92.3
MLR	≥1	77.7	82.9	78.1	82.6
PLR	≥345.9	79.5	67.9	66	80.9
NLR + MLR	≥0.25	90.9	84.3	82	92.2
NLR + PLR	≥0.28	90	85.7	83.2	91.9
NLR + PLR + MLR	≥0.29	90	86.1	83.5	91.6
MLR + NEU	≥0.25	93.2	86.1	84	94.1

**Table 4:** Correlations of NLR, MLR, PLR, and age with length of hospital stay.

Length of hospital stay	NLR	MLR	PLR	Age

*r* value	0.632	0.513	0.467	0.539
*p* value	0.000	0.000	0.000	0.000

## Data Availability

Data supporting this research article are available from the corresponding author on reasonable request.

## References

[1] Satici C., Demirkol M. A., Sargin Altunok E. (2020). Performance of pneumonia severity index and CURB-65 in predicting 30-day mortality in patients with COVID-19. *International Journal of Infectious Diseases*.

[2] Wu Z., McGoogan J. M. (2020). Characteristics of and important lessons from the coronavirus disease 2019 (COVID-19) outbreak in China summary of a report of 72 314 cases from the Chinese center for disease control and prevention. *Journal of the American Medical Association*.

[3] Zhao Q., Meng M., Kumar R. (2020). Lymphopenia is associated with severe coronavirus disease 2019 (COVID‐19) infections: a systemic review and meta‐analysis. *International Journal of Infectious Diseases*.

[4] Huang C., Wang Y., Li X. (2020). Clinical features of patients infected with 2019 novel coronavirus in Wuhan, China. *The Lancet*.

[5] Yang A.-P., Liu J.-p., Tao W.-q., Li H.-m. (2020). The diagnostic and predictive role of NLR, d-NLR and PLR in COVID-19 patients. *International Immunopharmacology*.

[6] Chen N., Zhou M., Dong X. (2020). Epidemiological and clinical characteristics of 99 cases of 2019 novel coronavirus pneumonia in Wuhan, China: a descriptive study. *The Lancet*.

[7] Qin C., Zhou L., Hu Z. (2020). Dysregulation of immune response in patients with COVID‐19 in Wuhan, China. *Clinical Infectious Diseases*.

[8] Mehta P., McAuley D. F., Brown M., Sanchez E., Tattersall R. S., Manson J. J. (2020). COVID-19: consider cytokine storm syndromes and immunosuppression. *The Lancet*.

[9] Lagunas‐Rangel F. A. (2020). Neutrophil‐to‐lymphocyte ratio and lymphocyte‐to‐C‐reactive protein ratio in patients with severe coronavirus disease 2019 (COVID‐19): a meta‐analysis. *Journal of Medical Virology*.

[10] Fan B. E., Chong V. C. L., Chan S. S. W. (2020). Hematologic parameters in patients with COVID-19 infection. *American Journal of Hematology*.

[11] Eyüboğlu F., Esendağlı D., Aktaş F. (2020). *COVID-19: Clinical and Laboratory Findings*.

[12] Huang Z., Fu Z., Huang W., Huang K. (2020). Prognostic value of neutrophil-to-lymphocyte ratio in sepsis: a meta-analysis. *The American Journal of Emergency Medicine*.

[13] Liu C.-C., Ko H.-J., Liu W.-S. (2019). Neutrophil-to-lymphocyte ratio as a predictive marker of metabolic syndrome. *Medicine*.

[14] Wang Q., Ma J., Jiang Z., Ming L. (2018). Prognostic value of neutrophil-to-lymphocyte ratio and platelet-to-lymphocyte ratio in acute pulmonary embolism: a systematic review and meta-analysis. *International Angiology*.

[15] Ethier J.-L., Desautels D., Templeton A., Shah P. S., Amir E. (2017). Prognostic role of neutrophil-tolymphocyte ratio in breast cancer: a systematic review and meta-analysis. *Breast Cancer Research*.

[16] Qu R., Ling Y., Zhang Y. h. z. (2020). Platelet‐to‐lymphocyte ratio is associated with prognosis in patients with coronavirus disease‐19. *Journal of Medical Virology*.

[17] Li B., Zhou P., Liu Y. (2018). Platelet-to-lymphocyte ratio in advanced Cancer: review and meta-analysis. *Clinica Chimica Acta*.

[18] Sun S., Cai X., Wang H. (2020). Abnormalities of peripheral blood system in patients with COVID-19 in Wenzhou, China. *Clinica Chimica Acta*.

[19] Ding R., Zhou X., Huang D. (2021). Predictive performances of blood parameter ratios for liver inflammation and advanced liver fibrosis in chronic hepatitis B infection. *BioMed Research International*.

[20] Erre G. L., Paliogiannis P., Castagna F. (2019). Meta‐analysis of neutrophil‐to‐lymphocyte and platelet‐to‐ lymphocyte ratio in rheumatoid arthritis. *European Journal of Clinical Investigation*.

[21] Ministry of Health (2020). *Guidance to COVID-19(SARSCov2infection)*.

[22] Hanson K. E., Caliendo A. M., Arias C. A. (2020). Infectious diseases society of America guidelines on the diagnosis of COVID-19. *Clinical Infectious Diseases*.

[23] Tang N., Li D., Wang X., Sun Z. (2020). Abnormal coagulation parameters are associated with poor prognosis in patients with novel coronavirus pneumonia. *Journal of Thrombosis and Haemostasis*.

[24] Zhu N., Zhang D., Wang W. (2020). Novel coronavirus from patients with pneumonia in China, 2019. *New England Journal of Medicine*.

[25] Xiang N., Havers F., Chen T. (2013). Use of national pneumonia surveillance to describe influenza A(H7N9) virus epidemiology, China,2004-2013. *Emerging Infectious Diseases*.

[26] Ponti G., Maccaferri M., Ruini C., Tomasi A., Ozben T. (2020). Biomarkers associated with COVID-19 disease progression. *Critical Reviews in Clinical Laboratory Sciences*.

[27] Xu Z., Shi L., Wang Y. (2020). Pathological findings of COVID-19 associated with acute respiratory distress syndrome. *The Lancet Respiratory Medicine*.

[28] Zhu J., Ji P., Pang J. (2020). Clinical characteristics of 3,062 COVID19 patients: a meta analysis. *Journal of Medical Virology*.

[29] Poissy J., Goutay J., Caplan M. (2020). Pulmonary embolism in COVID-19 patients: awareness of an increased prevalence. *Circulation*.

[30] Shi Y., Wang Y., Shao C. (2020). COVID-19 infection: the perspectives on immune responses. *Cell Death & Differentiation*.

[31] Henry B. M., de Oliveira M. H. S., Benoit S., Plebani M., Lippi G. (2020). Hematologic, biochemical and immune biomarker abnormalities associated with severe illness and mortality in coronavirus disease 2019 (COVID-19): a meta-analysis. *Clinical Chemistry and Laboratory Medicine*.

[32] Mo P., Xing Y., Xiao Y. (2020). Clinical characteristics of refractory COVID-19 pneumonia in Wuhan, China. *Clinical Infectious Diseases*.

[33] Akhmerov A., Marbán E. (2020). COVID-19 and the heart. *Circulation Research*.

[34] Chen T., Wu D., Chen H. (2020). Clinical characteristics of 113 deceased patients with coronavirus disease 2019: retrospective study. *BMJ*.

[35] Wang L., He W., Yu X. (2020). Coronavirus disease 2019 in elderly patients: characteristics and prognostic factors based on 4-week follow-up. *Journal of Infection*.

[36] Du R.-H., Liang L.-R., Yang C.-Q. (2020). Predictors of mortality for patients with COVID-19 pneumonia caused by SARS-CoV-2: a prospective cohort study. *European Respiratory Journal*.

[37] Haybar H., Pezeshki S. M. S., Saki N. (2019). Evaluation of complete blood count parameters in cardiovascular diseases: an early indicator of prognosis?. *Experimental and Molecular Pathology*.

[38] Channappanavar R., Perlman S. (2017). Pathogenic human coronavirus infections: causes and consequences of cytokine storm and immunopathology. *Seminars in Immunopathology*.

[39] Liu Y., Du X., Chen J. (2020). Neutrophil-to-lymphocyte ratio as an independent risk factor for mortality in hospitalized patients with COVID-19. *Journal of Infection*.

[40] Merad M., Martin J. C. (2020). Pathological inflammation in patients with COVID-19: a key role for monocytes and macrophages. *Nature Reviews Immunology*.

[41] Jenne C. N., Kubes P. (2015). Platelets in inflammation and infection. *Platelets*.

[42] Eickmann M., Gravemann U., Handke W. (2020). Inactivation of three emerging viruses-severe acute respiratory syndrome coronavirus, Crimean‐Congo haemorrhagic fever virus and Nipah virus ‐ in platelet concentrates by ultraviolet C light and in plasma by methylene blue plus visible light. *Vox Sanguinis*.

[43] Rayes J., Bourne J. H., Brill A., Watson S. P. (2019). The dual role of platelet‐ innate immune cell interactions in thrombo‐inflammation. *Research and Practice in Thrombosis and Haemostasis*.

[44] Gasparyan A. Y., Ayvazyan L., Mukanova U., Yessirkepov M., Kitas G. D. (2019). The platelet-to-lymphocyte ratio as an inflammatory marker in rheumatic diseases. *Annals of Laboratory Medicine*.

[45] Jimeno S., Ventura P. S., Castellano J. M. (2021). Prognostic implications of neutrophil-lymphocyte ratio in COVID-19. *European Journal of Clinical Investigation*.

